# Preclinical evaluation of JQ1 in esophageal squamous cell carcinoma: growth and migration suppression with BRD4-associated DNA damage and apoptotic signaling

**DOI:** 10.3389/fphar.2026.1829810

**Published:** 2026-09-03

**Authors:** Chunlan Pu, Jianyu Liu, Hengrui Fan, Huan Hu, Tao Chen, Jiao Tang

**Affiliations:** 1 Medical Research Center, The Affiliated Hospital of Southwest Jiaotong University, The Third People’s Hospital of Chengdu, Chengdu, Sichuan, China; 2 Department of Laboratory Medicine, Xindu District People’s Hospital, Chengdu, Sichuan, China

**Keywords:** alkaline comet assay, apoptosis, BET inhibitor, BRD4, cell migration, DNA damage response, esophageal squamous cell carcinoma, JQ1

## Abstract

**Background:**

Esophageal squamous cell carcinoma (ESCC) is an aggressive malignancy with limited therapeutic options. Bromodomain-containing protein 4 (BRD4), a BET family epigenetic reader, regulates oncogenic transcription and may represent a pharmacological vulnerability in ESCC. This study evaluated the pharmacological effects of the BET inhibitor JQ1 in ESCC models, with particular attention to cell migration under non-cytotoxic conditions and BRD4-associated stress signaling.

**Methods:**

The effects of JQ1 were examined in ESCC cell lines and in a KYSE150 subcutaneous xenograft model established in BALB/c nude mice. Cell viability was assessed by CCK-8 assays at 24, 48, and 72 h. Wound healing and Transwell assays were performed using cell-line-specific low concentrations of JQ1 that did not significantly reduce cell viability during the 24 h assay period, as confirmed by parallel viability controls. Cell-cycle distribution and apoptosis were analyzed by flow cytometry. Western blotting, immunofluorescence staining, qRT-PCR, and alkaline comet assay were used to assess BRD4-related signaling, apoptosis-associated proteins, γ-H2AX accumulation, and DNA strand breaks.

**Results:**

JQ1 reduced ESCC cell viability and clonogenic growth, induced G1-phase accumulation, and promoted apoptosis-associated changes. Under non-cytotoxic conditions, JQ1 reduced wound closure and Transwell migration in KYSE150, KYSE450, and ECA109 cells. Molecular analyses showed that JQ1 treatment was accompanied by decreased BRD4 expression, suppression of c-Myc/Cyclin D1-related signaling, increased p53 and Bax expression, reduced Bcl-2 expression, and elevated levels of cleaved PARP1 and cleaved Caspase-3. JQ1 also increased γ-H2AX accumulation and DNA strand breaks. *In vivo*, JQ1 suppressed KYSE150 xenograft growth and reduced Ki67 expression, while body weight monitoring and routine H&E staining did not reveal obvious toxicity under the current experimental conditions.

**Conclusion:**

JQ1 exhibited anti-proliferative, pro-apoptotic, and anti-migratory effects in preclinical ESCC models. These effects were accompanied by BRD4-related signaling changes, DNA damage-associated responses, and apoptosis-related molecular alterations. The findings support further investigation of BET/BRD4-associated pharmacological strategies in ESCC, while target-specific validation and expanded *in vivo* mechanistic and safety studies remain warranted.

## 1 Introduction

Esophageal squamous cell carcinoma (ESCC) is the predominant histological subtype of esophageal cancer worldwide and has a particularly high incidence in East Asia (Morgan et al., 2022). Although multimodal treatments, including surgical resection, neoadjuvant chemoradiotherapy, and immune checkpoint blockade, have improved clinical management, ESCC remains an aggressive malignancy with poor long-term outcomes (Kojima and Doi, 2017; Malhotra et al., 2019; Ooki et al., 2023). Many patients are diagnosed at a locally advanced stage, and recurrence, lymph node involvement, radiotherapy resistance, and distant metastasis continue to limit survival (Abnet et al., 2018; Puhr et al., 2023; Tsuji et al., 2023; Wang et al., 2023). These clinical challenges underscore the need to explore additional molecular vulnerabilities and therapeutic strategies for ESCC.

Bromodomain-containing protein 4 (BRD4), a member of the bromodomain and extra-terminal domain (BET) family, functions as an epigenetic reader that recognizes acetylated lysine residues on histones and regulates transcriptional programs involved in cell-cycle progression, DNA replication, and oncogenic signaling (Devaiah et al., 2024; Stathis and Bertoni, 2018; Taniguchi, 2016). JQ1 is a small-molecule BET bromodomain inhibitor widely used to investigate BET-dependent transcriptional regulation in cancer models (Ding et al., 2025; Garcia et al., 2018; Wang et al., 2019). Previous studies have shown that JQ1 can suppress oncogenic transcriptional programs, including c-Myc-related signaling, and exert anti-proliferative or pro-apoptotic effects in multiple tumor types (Pu et al., 2024; Shahbazi et al., 2016; Wu et al., 2020).

In ESCC, BRD4 upregulation has been associated with tumor progression and poor prognosis (Efe et al., 2023; Li et al., 2023; Wu et al., 2022). Pharmacological studies have also suggested that JQ1 inhibits ESCC cell proliferation, at least in part, through suppression of the Cyclin D1/c-Myc axis (Ding et al., 2023; Wang et al., 2018). However, the effects of BET/BRD4 inhibition on ESCC cell migration appear to be context-dependent. For example, BRD4 inhibition has been reported to enhance ESCC cell migration through autophagy- and EMT-related mechanisms (Yang et al., 2022), whereas other tumor models have shown migration-suppressive effects of JQ1. This inconsistency highlights the importance of evaluating JQ1-induced changes in cell motility under conditions that minimize cytotoxicity-related confounding.

The present study systematically evaluated the pharmacological effects of JQ1 in ESCC experimental models. We assessed cell viability, clonogenic growth, wound closure, Transwell migration, cell-cycle progression, apoptosis, and xenograft growth. To strengthen the interpretation of migration-related findings, wound healing and Transwell assays were performed using cell-line-specific low concentrations that did not significantly reduce cell viability during the assay period. We further examined BRD4-related signaling, apoptosis-associated proteins, γ-H2AX accumulation, and DNA strand breaks using Western blotting, immunofluorescence, qRT-PCR, and alkaline comet assay. This study provides a controlled preclinical evaluation of JQ1 in ESCC and identifies BRD4-associated stress and apoptotic signaling as candidate mechanisms that require further validation.

## 2 Materials and methods

### 2.1 Reagents and antibodies

JQ1 was obtained from MedChemExpress. JQ1 was dissolved in dimethyl sulfoxide (DMSO) to prepare a 20 mM stock solution, which was aliquoted and stored at −80 °C until use. Antibodies used in the present study were purchased from Proteintech Group, Inc. and Cell Signaling Technology, Inc. Primary antibodies were diluted according to the manufacturers’ recommendations and are listed in Table 1.

**TABLE 1:** Antibodies and working dilutions used in this study.

Antibody	Host	Company	Catalog no.	Dilution	Application
BRD4	Mouse	CST	63759	1:1,000 (WB) 1:200 (IF)	WB/IF
c-Myc	Rabbit	Proteintech	10828-1-AP	1:1,000	WB
Cyclin D1	Mouse	Proteintech	60186-1-Ig	1:5,000	WB
CDK4	Rabbit	Proteintech	11026-1-AP	1:2,000	WB
CDK6	Rabbit	Proteintech	14052-1-AP	1:2,000	WB
p53	Rabbit	Proteintech	10442-1-AP	1:5,000	WB
γ-H2AX	Rabbit	CST	9718	1:1,000 (WB) 1:300 (IF)	WB/IF
Bax	Rabbit	Proteintech	50599-2-Ig	1:2,000	WB
Bcl-2	Rabbit	Proteintech	12789-1-AP	1:2,000	WB
β-actin	Mouse	Proteintech	66009-1-Ig	1:20,000	WB
Secondary antibody	Mouse	Proteintech	SA00001-1	1:5,000	WB
Secondary antibody	Rabbit	Proteintech	RGAR001	1:5,000	WB
Ki67	Rabbit	Proteintech	27309-1-AP	1:4,000	IHC

### 2.2 Cell lines and cell culture

The human ESCC cell lines KYSE150, KYSE450, and ECA109, the human esophageal adenocarcinoma (EAC) cell line OE19, and the normal human esophageal epithelial cell line Het-1A were obtained from Procell Life Science and Technology Co., Ltd. and maintained in our laboratory. Cells were cultured in RPMI-1640 medium (Gibco; Thermo Fisher Scientific, Inc.) supplemented with 10% fetal bovine serum (FBS; Gibco; Thermo Fisher Scientific, Inc.) and 1% penicillin-streptomycin at 37 °C in a humidified atmosphere containing 5% CO_2_. The medium was replaced every 3 days. When cultures reached approximately 90% confluence, cells were detached with 0.25% trypsin and passaged at a ratio of 1:3. All cell lines were authenticated by short tandem repeat (STR) profiling and verified to be mycoplasma-free before use.

### 2.3 Cell viability assay

Cell viability was evaluated using the Cell Counting Kit-8 (CCK-8) assay. Cells were seeded into 96-well plates at a density of 3–5 × 10^3^ cells/well and incubated overnight to allow adherence. Cells were then treated with increasing concentrations of JQ1 (0–80 μM) for 24, 48, or 72 h at 37 °C with 5% CO_2_. After treatment, optical density at 450 nm was measured according to the manufacturer’s protocol (Beyotime Biotechnology).

### 2.4 Cell colony formation assay

ESCC cells were seeded into 6-well plates at a density of 800–1,000 cells/well and incubated overnight to allow adherence. Cells were then treated with JQ1 (0–10 μM) or vehicle control (0.1% DMSO) for 10 days. After treatment, cells were gently washed with phosphate-buffered saline (PBS), fixed with 4% paraformaldehyde at room temperature for 15 min, and stained with 0.5% crystal violet at room temperature for 15 min. Images of stained colonies were captured, and colonies containing at least 50 cells were counted under a light microscope. Colony numbers were calculated from three independent experiments.

### 2.5 Wound healing assay

Cells were seeded into 6-well plates at a density of 3 × 10^5^ cells/well and cultured until they formed a confluent monolayer of approximately 80%–90% confluence. A straight scratch was created in the monolayer using a sterile 200 μL pipette tip, and detached cells were removed by washing with PBS. To minimize the potential confounding effects of cytotoxicity or proliferation inhibition, wound healing assays were performed using cell-line-specific low concentrations of JQ1. KYSE150 and KYSE450 cells were treated with DMSO or JQ1 at 0.05, 0.10, and 0.20 μM, whereas ECA109 cells were treated with DMSO or JQ1 at 0.25, 0.50, and 1.0 μM. Treatments were performed in serum-free medium for 24 h at 37 °C in a humidified atmosphere containing 5% CO2. Bright-field images were captured from randomly selected regions at 0 and 24 h using a light microscope (Leica) at ×10 magnification. Wound width was measured using ImageJ software (version 1.46r, National Institutes of Health). Wound closure rate was calculated as follows: Wound closure rate (%) = [(initial wound width − mean remaining wound width)/initial wound width] × 100. Cell viability was assessed under the same treatment conditions and duration to confirm that the selected concentrations did not significantly affect cell survival during the assay period.

### 2.6 Transwell migration assay

Cell migration was evaluated using 24-well Transwell chambers with 8 μm pore membranes (Costar; Corning, Inc.). A total of 5 × 10^4^ cells in 200 μL serum-free medium were seeded into the upper chamber, and 600 μL medium containing 10% FBS was added to the lower chamber as a chemoattractant. To reduce cytotoxicity-related interference, Transwell migration assays were performed using the same cell-line-specific low concentrations of JQ1 as those used in the wound healing assay. After 24 h of incubation at 37 °C with 5% CO_2_, non-migrated cells on the upper surface of the membrane were carefully removed. Migrated cells on the lower surface were fixed with 4% paraformaldehyde at room temperature for 15 min and stained with 0.5% crystal violet at room temperature for 15 min. Migrated cells were counted in five randomly selected fields under a light microscope (Leica). Cell viability was evaluated in parallel under the same treatment conditions and duration to verify that the selected JQ1 concentrations did not significantly affect cell survival during the migration assay.

### 2.7 Flow cytometry

Cell-cycle distribution and apoptosis were analyzed by flow cytometry. ESCC cells were seeded in 6-well plates at a density of 3 × 10^5^ cells/well and incubated at 37 °C with 5% CO_2_ for 24 h. Cells were then treated with JQ1 at the indicated concentrations for 24 h for cell-cycle analysis or for 48 h for apoptosis analysis; 0.1% DMSO was used as the vehicle control. For cell-cycle analysis, cells were harvested and fixed in ice-cold 70% ethanol at 4 °C overnight. After fixation, cells were washed twice with cold PBS and incubated with DNase-free RNase and propidium iodide (PI) according to the manufacturer’s instructions (Cell Cycle Detection Kit; cat. no. KGA512; Jiangsu KeyGen Biotech Co., Ltd.).

For apoptosis analysis, ethanol fixation was omitted. Cells were stained with Annexin V-FITC and PI using the Annexin V-FITC/PI Apoptosis Detection Kit (cat. no. KGA107; Jiangsu KeyGen Biotech Co., Ltd.) for 15–30 min at room temperature in the dark according to the manufacturer’s protocol. Flow cytometric analysis was performed using a CytoFLEX flow cytometer (Beckman Coulter, Inc.). PI fluorescence was detected using the PE-A channel (585/42 nm) to optimize signal separation from the FITC channel. Cell-cycle distribution was analyzed using ModFit LT software (version 5.0; Verity Software House), and apoptosis data were processed using CytExpert software (version 2.4; Beckman Coulter, Inc.).

### 2.8 Western blot analysis

Cells were seeded in 6-well plates at a density of 3–5 × 10^5^ cells/well and treated with the indicated concentrations of JQ1 for 24 h at 37 °C with 5% CO_2_. Treated and control cells were collected and lysed on ice for 30 min in RIPA buffer (Solarbio Science and Technology Co., Ltd.) supplemented with 1% protease inhibitor cocktail and 1% PMSF. Cell lysates were centrifuged at 13,000 ×g for 15 min at 4 °C, and the supernatants were collected. Total protein concentrations were determined using the BCA Protein Assay Kit (Thermo Fisher Scientific, Inc.). Equal amounts of protein (20–30 μg) were separated by SDS-PAGE using 8%, 10%, 4%–12%, or 4%–20% gels and transferred onto PVDF membranes (MilliporeSigma). Membranes were blocked with 5% non-fat milk in TBS-Tween 20 for 1.5 h at room temperature and incubated with primary antibodies overnight at 4 °C. After washing with TBS-T, membranes were incubated with HRP-conjugated secondary antibodies for 1–2 h at room temperature. Protein bands were visualized using an enhanced chemiluminescence detection system (MilliporeSigma) and a contact-based non-destructive quantitative imaging system (YiboTech Life Sciences, Shanghai, China). Protein expression levels were semi-quantified using ImageJ software and normalized to the corresponding loading control.

### 2.9 Immunofluorescence staining

ESCC cells were seeded onto 14 mm glass coverslips placed in 24-well plates at a density of 3 × 10^3^ cells/well and incubated overnight to allow attachment. Cells were then treated with the indicated concentrations of JQ1 for 24 h at 37 °C with 5% CO_2_, with 0.1% DMSO serving as the vehicle control. After treatment, cells were rinsed three times with cold PBS and fixed with 4% paraformaldehyde at room temperature for 15 min. Cells were permeabilized with 0.4% Triton X-100 for 15 min and blocked with 5% bovine serum albumin (Solarbio Science and Technology Co., Ltd.) for 30 min at room temperature. Cells were incubated overnight at 4 °C with primary antibodies against BRD4 (1:200) and γ-H2AX (1:300). After washing, cells were incubated with CoraLite488-conjugated goat anti-mouse IgG (H + L) (cat. no. SA00013-1) and rhodamine-conjugated goat anti-rabbit IgG (cat. no. SA00007-2) secondary antibodies (1:250; Proteintech Group, Inc.) for 1 h at room temperature in the dark. Nuclei were counterstained with DAPI, and images were captured using a fluorescence microscope (Leica Microsystems GmbH).

### 2.10 Alkaline comet assay

DNA strand breaks were detected using a Comet Assay Kit (3-Well Slides; Abbkine, Cat. No. KTA3040) according to the manufacturer’s protocol. Briefly, KYSE150, KYSE450, and ECA109 cells were treated with DMSO or JQ1 at 0.5 μM for 24 h. After treatment, cells were collected, washed with cold PBS, and resuspended at a density of 1–5 × 10^5^ cells/mL. The cell suspension was mixed with low-melting-point agarose at 37 °C and loaded onto 3-well comet slides. After agarose solidification at 4 °C in the dark, slides were immersed in precooled lysis buffer and then incubated in alkaline electrophoresis solution to allow DNA unwinding. Electrophoresis was performed in 1× alkaline electrophoresis solution at 25 V for 20 min. Slides were washed with deionized water and stained with propidium iodide in the dark. Comet images were captured using a fluorescence microscope and analyzed with comet assay analysis software. At least 50 randomly selected cells were analyzed for each sample, and DNA damage was quantified as the percentage of DNA in the comet tail.

### 2.11 *In vivo* tumor xenograft model

All animal procedures were approved by the Experimental Animal Management Committee of Southwest Jiaotong University (Chengdu, China; approval no. SWJTU-2503-NSFC-135) and conducted in accordance with institutional animal care guidelines. Female BALB/c nude mice (5–6 weeks old, approximately 18 g; Beijing HFK Bioscience Co., Ltd.) were used to establish the subcutaneous tumor model. Mice were acclimated for 1 week with free access to food and water under controlled conditions (22 °C–26 °C, 50%–65% relative humidity, 12 h light/dark cycle). For xenograft establishment, 3 × 10^6^ KYSE150 cells suspended in 50% Matrigel (Corning, Inc.; cat. no. 356237) were subcutaneously injected into the right flank of each mouse. Once tumors became palpable, mice were randomly assigned to the vehicle control, JQ1 (20 mg/kg), or JQ1 (40 mg/kg) group. Treatments were administered by intraperitoneal injection. Tumor volume and body weight were monitored periodically. Tumor volume was calculated as V = 0.5 × length × width^2^. At the end of the experiment, tumors and major organs were excised, weighed, and fixed for subsequent analysis. Tumor growth inhibition (TGI) was calculated based on tumor weights.

### 2.12 Immunohistochemistry and H&E staining

Excised tumors and major organs, including the lung, liver, spleen, kidney, and heart, were fixed in 4% paraformaldehyde, embedded in paraffin, and sectioned. For histopathological assessment, organ sections were stained with hematoxylin and eosin (H&E). For immunohistochemistry, tumor sections were incubated with an anti-Ki67 primary antibody (cat. no. 27309-1-AP; 1:4,000), followed by an appropriate secondary antibody. Positive staining was visualized using DAB, and the percentage of Ki67-positive cells was quantified under a microscope.

### 2.13 Quantitative real-time PCR (qRT-PCR)

KYSE150, KYSE450, and ECA109 cells were treated with DMSO or JQ1 at the indicated concentrations for 24 h. Total RNA was extracted from treated cells, and RNA concentration and purity were assessed before reverse transcription. Complementary DNA (cDNA) was synthesized using TransScript® All-in-One First-Strand cDNA Synthesis SuperMix for qPCR (TransGen Biotech, Cat. No. AT341) according to the manufacturer’s instructions. Quantitative real-time PCR was performed using Hieff® qPCR SYBR Green Master Mix (No Rox; Yeasen, Shanghai, China) on a real-time PCR detection system. Relative BRD4 mRNA expression was normalized to 18S rRNA and calculated using the 2^−ΔΔCT^ method. The primer sequences used for qRT-PCR were as follows: BRD4 forward, 5′-CGC​TAT​GTC​ACC​TCC​TGT​TTG​C-3′ and reverse, 5′-ACT​CTG​AGG​ACG​AGA​AGC​CCT​T-3′; 18S forward, 5′-CGG​CTA​CCA​CAT​CCA​AGG​AA-3′ and reverse, 5′-GCT​GGA​ATT​ACC​GCG​GCT-3′.

### 2.14 Statistical analysis

All data are presented as the mean ± SD from three independent biological experiments unless otherwise stated. Comparisons between two groups were performed using an unpaired two-tailed Student’s t-test. Comparisons among three or more groups were analyzed using one-way ANOVA followed by Tukey’s multiple comparisons test. Tumor growth curves and body weight curves were analyzed using two-way repeated-measures ANOVA followed by Tukey’s multiple comparisons test. A P value <0.05 was considered statistically significant (*P < 0.05, **P < 0.01, ***P < 0.001, ****P < 0.0001). Statistical analyses were conducted using GraphPad Prism version 8.0 (Dotmatics).

## 3 Results

### 3.1 JQ1 reduces ESCC cell viability in a time- and dose-dependent manner

**FIGURE 1:**
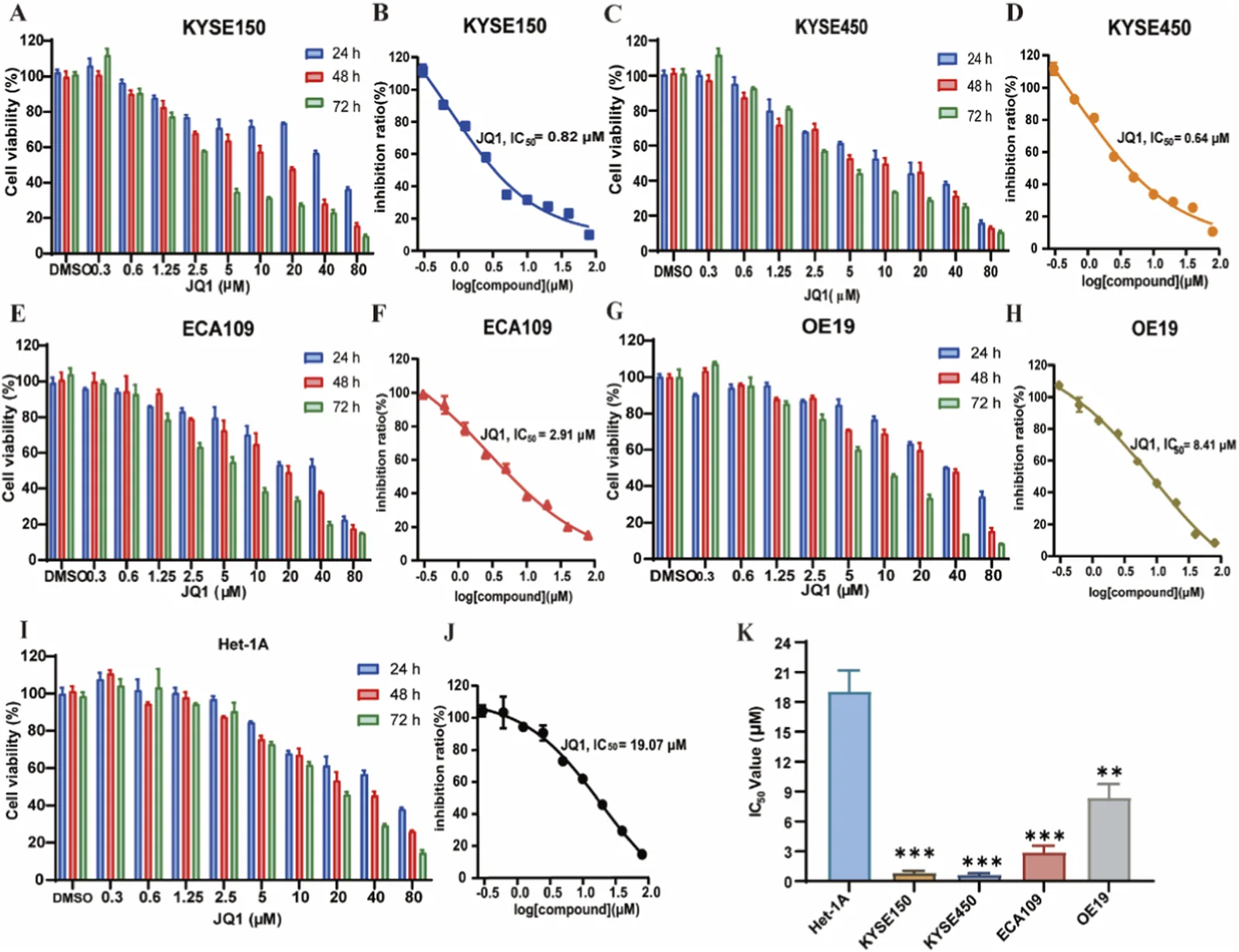
JQ1 reduces esophageal cancer cell viability in a dose- and time-dependent manner. **(A,C,E,G,I)** Cell viability of KYSE150, KYSE450, ECA109, OE19, and Het-1A cells following treatment with increasing concentrations of JQ1 for 24, 48, and 72 h, as determined by CCK-8 assay. **(B,D,F,H,J)** Dose-response curves and IC50 values calculated from the 72 h treatment. **(K)** Comparison of IC50 values among Het-1A and esophageal cancer cell lines following 72 h of JQ1 treatment. Data are presented as the mean ± SD from three independent biological experiments. Statistical significance was assessed by one-way ANOVA followed by Tukey’s multiple comparisons test. **P < 0.01 and ***P < 0.001 vs. Het-1A.

To evaluate the growth-inhibitory effect of JQ1, KYSE150, KYSE450, ECA109, OE19, and Het-1A cells were treated with increasing concentrations of JQ1 for 24, 48, and 72 h, followed by CCK-8 assay. As shown in Figures 1A–J, JQ1 reduced the viability of esophageal cancer cell lines in a concentration- and time-dependent manner, with greater inhibition observed after prolonged exposure. Compared with the 24 h treatment, the inhibitory effect was enhanced after 48 h and was most evident after 72 h in tumor cell lines. To further characterize the time-dependent response, IC50 values were calculated from the 72 h dose-response curves. The IC_50_ values were 0.82 ± 0.23 μM for KYSE150, 0.64 ± 0.15 μM for KYSE450, 2.91 ± 0.64 μM for ECA109, and 8.41 ± 1.35 μM for OE19 (Figures 1B,D,F,H). In contrast, the normal esophageal epithelial cell line Het-1A showed lower sensitivity to JQ1, with an IC_50_ value of 19.07 ± 2.11 μM (Figures 1I–K).

Comparison of IC_50_ values showed that KYSE150, KYSE450, ECA109, and OE19 cells had significantly lower IC_50_ values than Het-1A cells (Figure 1K). These findings indicate that esophageal cancer cells, particularly ESCC cell lines, were more sensitive to JQ1 than normal esophageal epithelial cells under the tested *in vitro* conditions. KYSE150 and KYSE450 were considered relatively sensitive models, whereas ECA109 was retained as a less-sensitive comparator in subsequent experiments.

### 3.2 JQ1 suppresses clonogenic growth of ESCC cells

**FIGURE 2:**
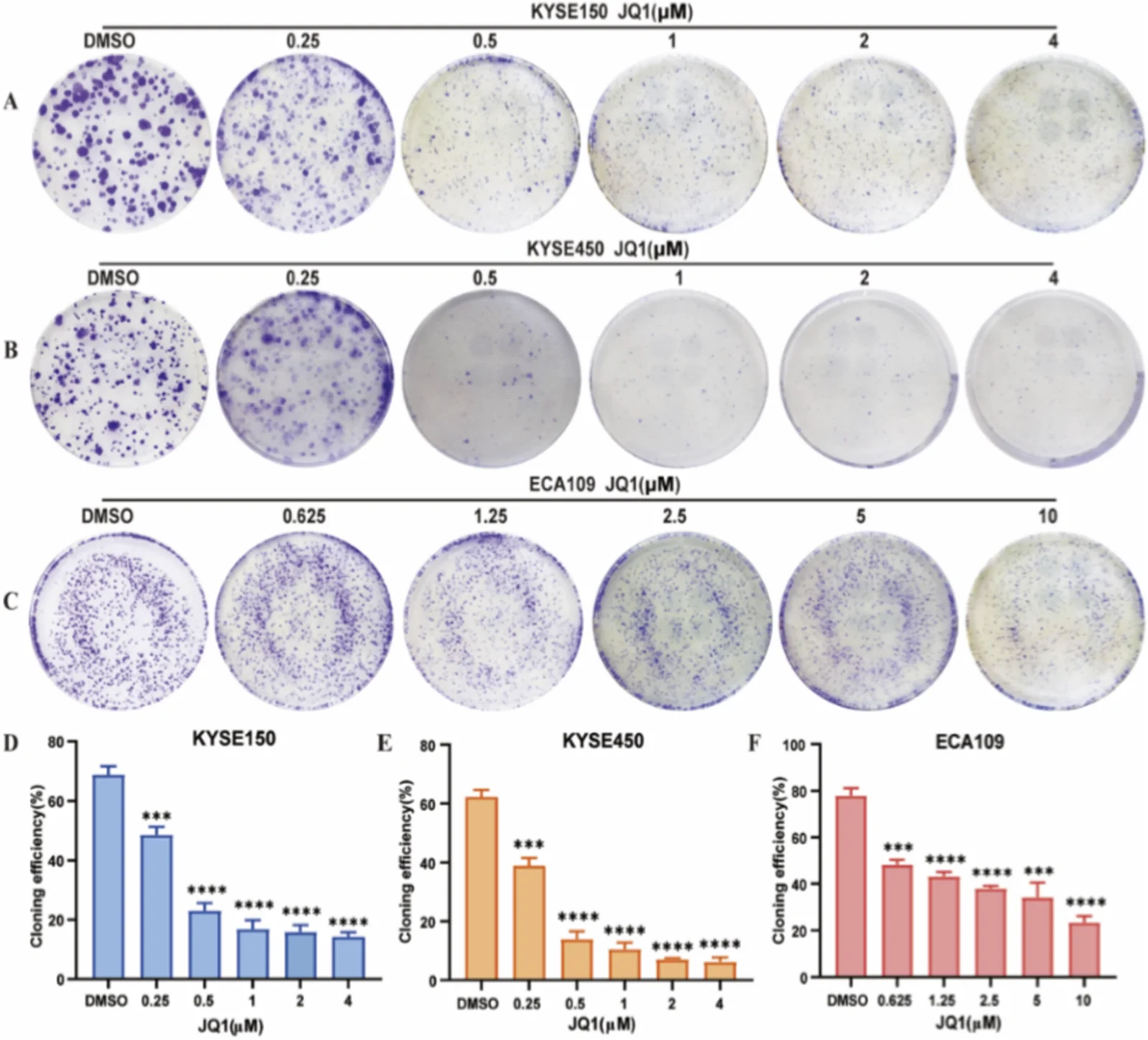
JQ1 suppresses clonogenic growth in ESCC cell lines. **(A)** Representative images of colony formation assays in KYSE150 cells. **(B)** Representative images of colony formation assays in KYSE450 cells. **(C)** Representative images of colony formation assays in ECA109 cells. Cells were treated with JQ1 at the indicated concentrations (0–10 μM) or vehicle control (DMSO) for 10 days. **(D)** Quantitative analysis of colony formation in KYSE150 cells. **(E)** Quantitative analysis of colony formation in KYSE450 cells. **(F)** Quantitative analysis of colony formation in ECA109 cells. Data are presented as the mean ± SD from three independent biological experiments. Statistical significance was assessed by one-way ANOVA followed by Tukey’s multiple comparisons test. ***P < 0.001, ****P < 0.0001 vs. DMSO control.

Given the inhibitory effect of JQ1 on ESCC cell viability, colony formation assays were performed to evaluate its effect on clonogenic growth. After 10 days of treatment, JQ1 reduced colony formation in KYSE150, KYSE450, and ECA109 cells in a concentration-dependent manner (Figures 2A–C). KYSE150 and KYSE450 cells showed greater reductions in colony formation at relatively lower concentrations, whereas ECA109 cells required higher concentrations to show comparable clonogenic inhibition. Quantitative analysis confirmed significant decreases in colony formation after JQ1 treatment in all three ESCC cell lines (Figures 2D–F). These results support the inhibitory effect of JQ1 on ESCC clonogenic growth.

### 3.3 JQ1 suppresses ESCC cell migration under non-cytotoxic conditions

**FIGURE 3:**
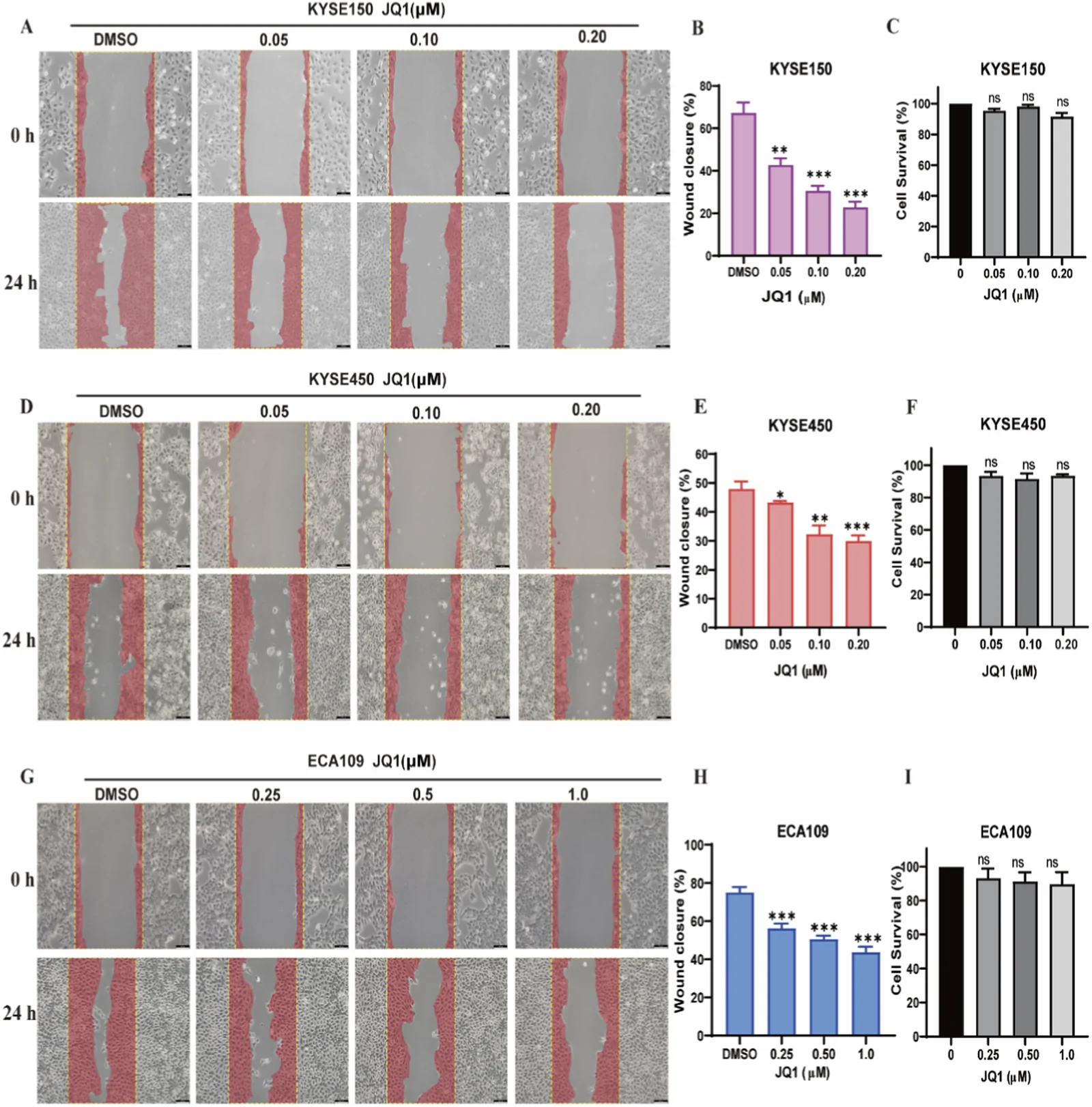
JQ1 suppresses wound closure in ESCC cells under non-cytotoxic conditions. **(A)** Representative wound healing images of KYSE150 cells treated with DMSO or JQ1 at 0.05, 0.10, and 0.20 μM for 24 h. **(B)** Quantification of wound closure rates in KYSE150 cells. **(C)** Cell survival of KYSE150 cells under the same treatment conditions. **(D)** Representative wound healing images of KYSE450 cells treated with DMSO or JQ1 at 0.05, 0.10, and 0.20 μM for 24 h. **(E)** Quantification of wound closure rates in KYSE450 cells. **(F)** Cell survival of KYSE450 cells under the same treatment conditions. **(G)** Representative wound healing images of ECA109 cells treated with DMSO or JQ1 at 0.25, 0.50, and 1.0 μM for 24 h. **(H)** Quantification of wound closure rates in ECA109 cells. **(I)** Cell survival of ECA109 cells under the same treatment conditions. Data are presented as the mean ± SD from three independent biological experiments. Statistical significance was assessed by one-way ANOVA followed by Tukey’s multiple comparisons test. *P < 0.05, **P < 0.01, ***P < 0.001, ****P < 0.0001; ns, not significant vs. DMSO control. Scale bar = 100 μm.

**FIGURE 4:**
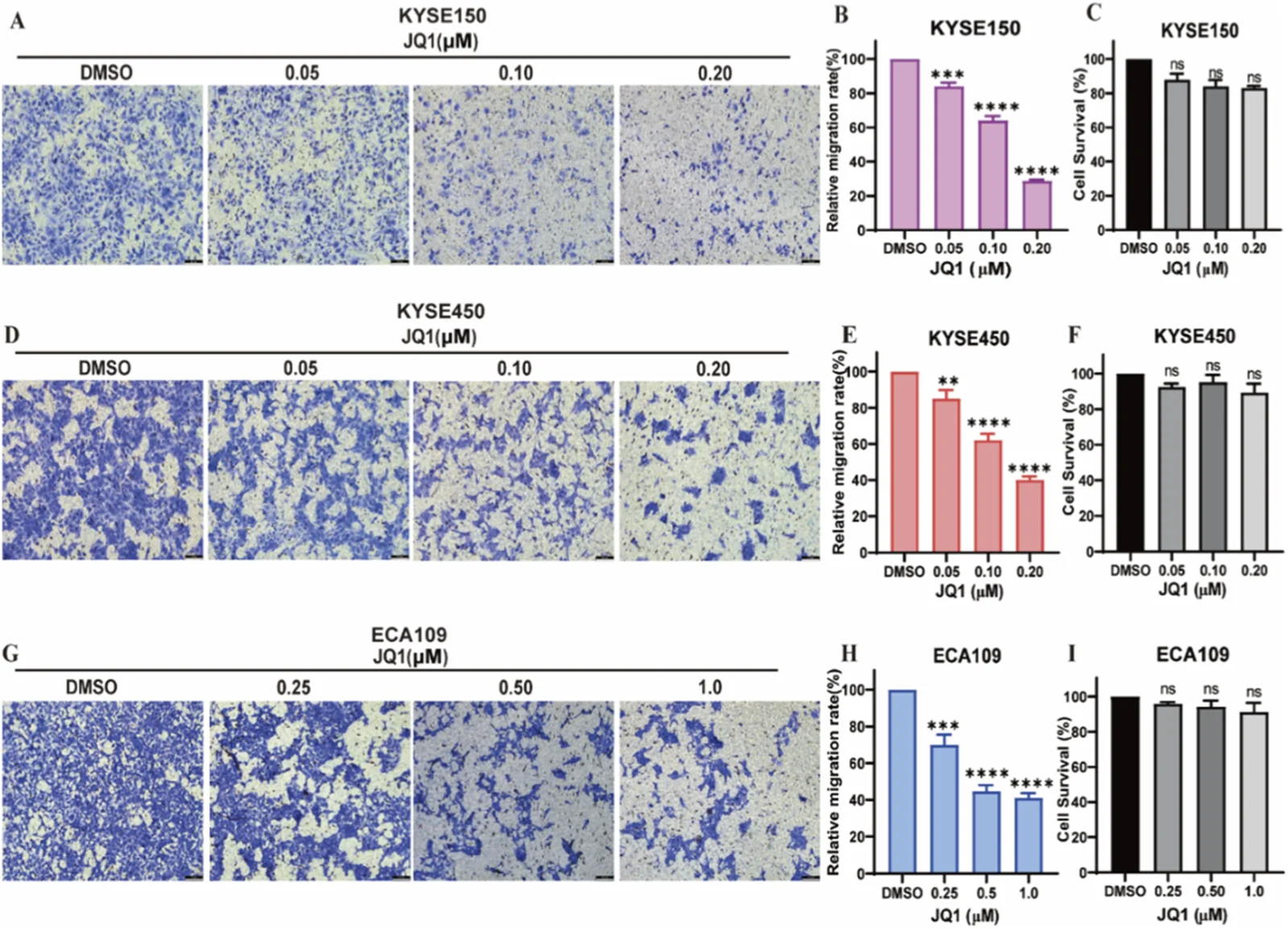
Transwell assays show the inhibitory effect of JQ1 on ESCC cell migration under non-cytotoxic conditions. **(A)** Representative images of migrated KYSE150 cells treated with DMSO or JQ1 at 0.05, 0.10, and 0.20 μM for 24 h. **(B)** Quantification of relative migration rates in KYSE150 cells. **(C)** Cell survival of KYSE150 cells under the same treatment conditions. **(D)** Representative images of migrated KYSE450 cells treated with DMSO or JQ1 at 0.05, 0.10, and 0.20 μM for 24 h. **(E)** Quantification of relative migration rates in KYSE450 cells. **(F)** Cell survival of KYSE450 cells under the same treatment conditions. **(G)** Representative images of migrated ECA109 cells treated with DMSO or JQ1 at 0.25, 0.50, and 1.0 μM for 24 h. **(H)** Quantification of relative migration rates in ECA109 cells. **(I)** Cell survival of ECA109 cells under the same treatment conditions. Data are presented as the mean ± SD from three independent biological experiments. Statistical significance was assessed by one-way ANOVA followed by Tukey’s multiple comparisons test. **P < 0.01, ***P < 0.001, ****P < 0.0001; ns, not significant vs. DMSO control. Scale bar = 100 μm.

Wound healing and Transwell assays (Grada et al., 2017; Justus et al., 2014) were used as complementary approaches to evaluate ESCC cell migration. To minimize potential confounding by cytotoxicity or proliferation inhibition, both assays were performed using cell-line-specific low concentrations of JQ1, with parallel viability controls under the same 24 h treatment conditions (Figures 3, 4).

In the wound healing assay, JQ1 reduced wound closure in KYSE150, KYSE450, and ECA109 cells after 24 h of treatment (Figure 3). In KYSE150 and KYSE450 cells, JQ1 at 0.05, 0.10, and 0.20 μM progressively decreased wound closure compared with the DMSO control (Figures 3A,B,D,E). Because ECA109 cells showed lower sensitivity to JQ1, a higher but non-cytotoxic concentration range was used; JQ1 at 0.25, 0.50, and 1.0 μM reduced wound closure in ECA109 cells (Figures 3G,H). Parallel viability assays showed no significant reduction in cell survival at these concentrations during the 24 h assay period (Figures 3C,F,I), indicating that impaired wound closure was not attributable to overt short-term cytotoxicity.

Transwell migration assays were then performed using the same cell-line-specific low concentrations. JQ1 reduced the number of migrated KYSE150 cells at 0.05, 0.10, and 0.20 μM, with the strongest inhibition observed at 0.20 μM (Figures 4A,B). Similar reductions were observed in KYSE450 cells across the 0.05–0.20 μM concentration range (Figures 4D,E). In ECA109 cells, JQ1 treatment at 0.25, 0.50, and 1.0 μM decreased relative migration rates compared with the DMSO group (Figures 4G,H). Consistent with the wound healing assay, parallel viability assays showed no significant changes in cell survival under these treatment conditions (Figures 4C,F,I).

Collectively, these results show that JQ1 reduces ESCC cell migration under non-cytotoxic conditions. The concordant findings from wound healing and Transwell assays indicate that the reduced migratory capacity is unlikely to be solely explained by short-term loss of cell viability.

### 3.4 JQ1 induces G1-phase cell cycle arrest in ESCC cells

**FIGURE 5:**
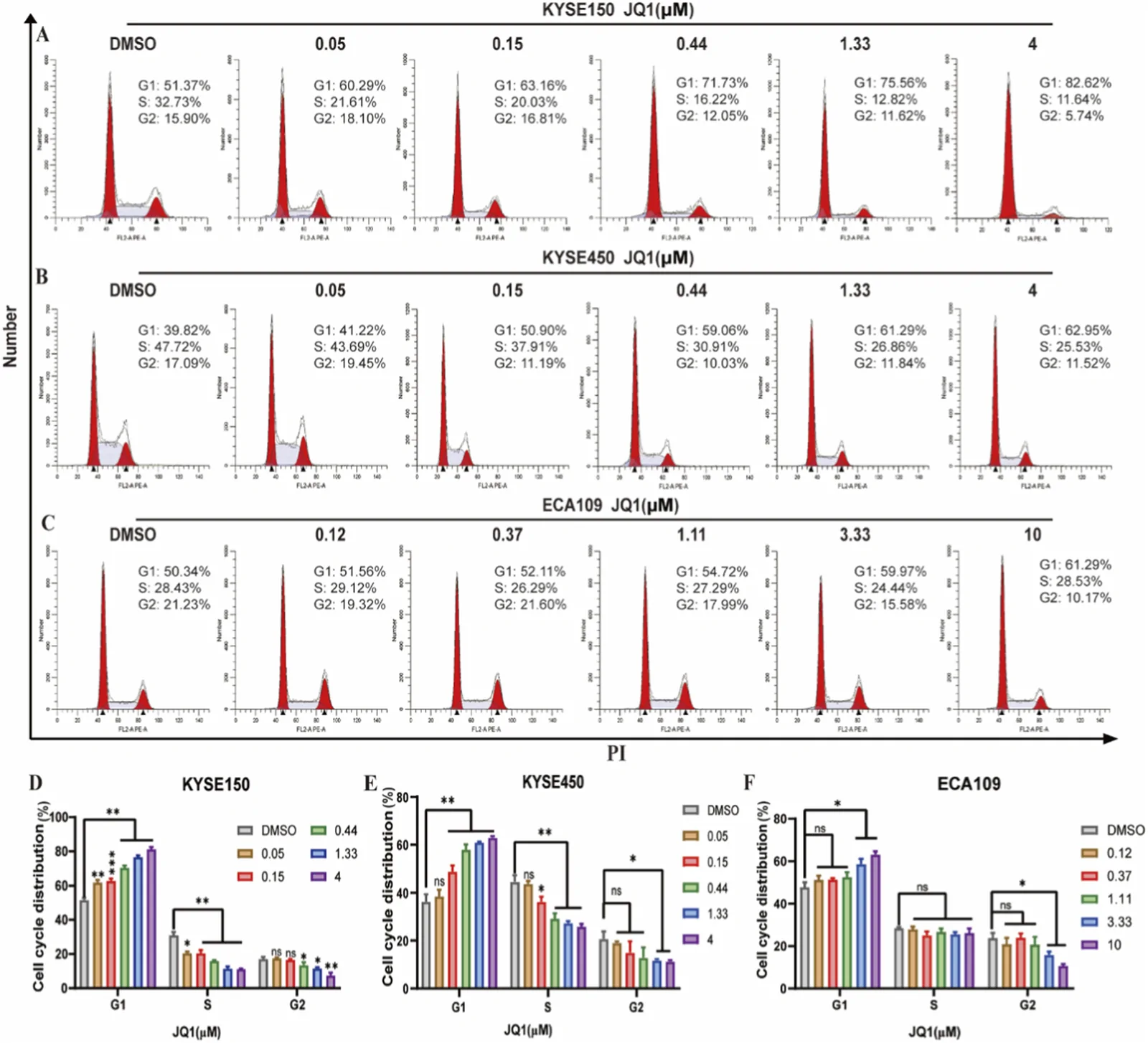
JQ1 induces G1-phase cell-cycle arrest in ESCC cells. **(A)** Representative flow cytometry plots showing cell-cycle distribution in KYSE150 cells. **(B)** Representative flow cytometry plots showing cell-cycle distribution in KYSE450 cells. **(C)** Representative flow cytometry plots showing cell-cycle distribution in ECA109 cells. Cells were treated with JQ1 at the indicated concentrations (0–10 μM) or vehicle control (DMSO) for 24 h. **(D)** Quantification of cell-cycle distribution in KYSE150 cells. **(E)** Quantification of cell-cycle distribution in KYSE450 cells. **(F)** Quantification of cell-cycle distribution in ECA109 cells. Data are presented as the mean ± SD from three independent biological experiments. Statistical significance was assessed by one-way ANOVA followed by Tukey’s multiple comparisons test. *P < 0.05, **P < 0.01, ***P < 0.001, ****P < 0.0001; ns, not significant vs. DMSO control.

Flow cytometry was performed to determine whether JQ1 affected cell-cycle distribution in ESCC cells. JQ1 treatment increased the proportion of cells in the G1 phase in a concentration-dependent manner, accompanied by reductions in the S and/or G2/M phase populations (Figure 5). In KYSE150 cells, JQ1 increased the G1-phase population by approximately 8.92% at 0.05 μM and by 30.89% at 4 μM, accompanied by a 21.09% reduction in the S-phase population and a decrease in the G2/M-phase population to 5.74% (Figures 5A,D). In KYSE450 cells, G1-phase accumulation was observed from 0.44 μM JQ1 and reached a 23.13% increase at 4 μM, together with reductions in the S- and G2/M-phase populations (Figures 5B,E). In ECA109 cells, marked G1-phase accumulation was observed mainly at concentrations above 3.33 μM, consistent with the lower sensitivity of this cell line (Figures 5C,F). These data indicate that JQ1 induces G1-phase accumulation in ESCC cells in a concentration- and cell line-dependent manner, consistent with the anti-proliferative effects observed in the preceding viability and colony formation assays.

### 3.5 JQ1 modulates BRD4-associated c-Myc/cyclin D1/CDK4/6 signaling in ESCC cells

Because JQ1 induced G1-phase accumulation, we examined BRD4 and several G1/S transition-related proteins by Western blotting. BRD4 is an epigenetic regulator involved in transcriptional control of cell-cycle genes, and c-Myc is one of the well-characterized downstream transcriptional programs linked to BRD4 activity (Niu et al., 2025; Pang et al., 2022; Wang L. et al., 2017).

**FIGURE 6:**
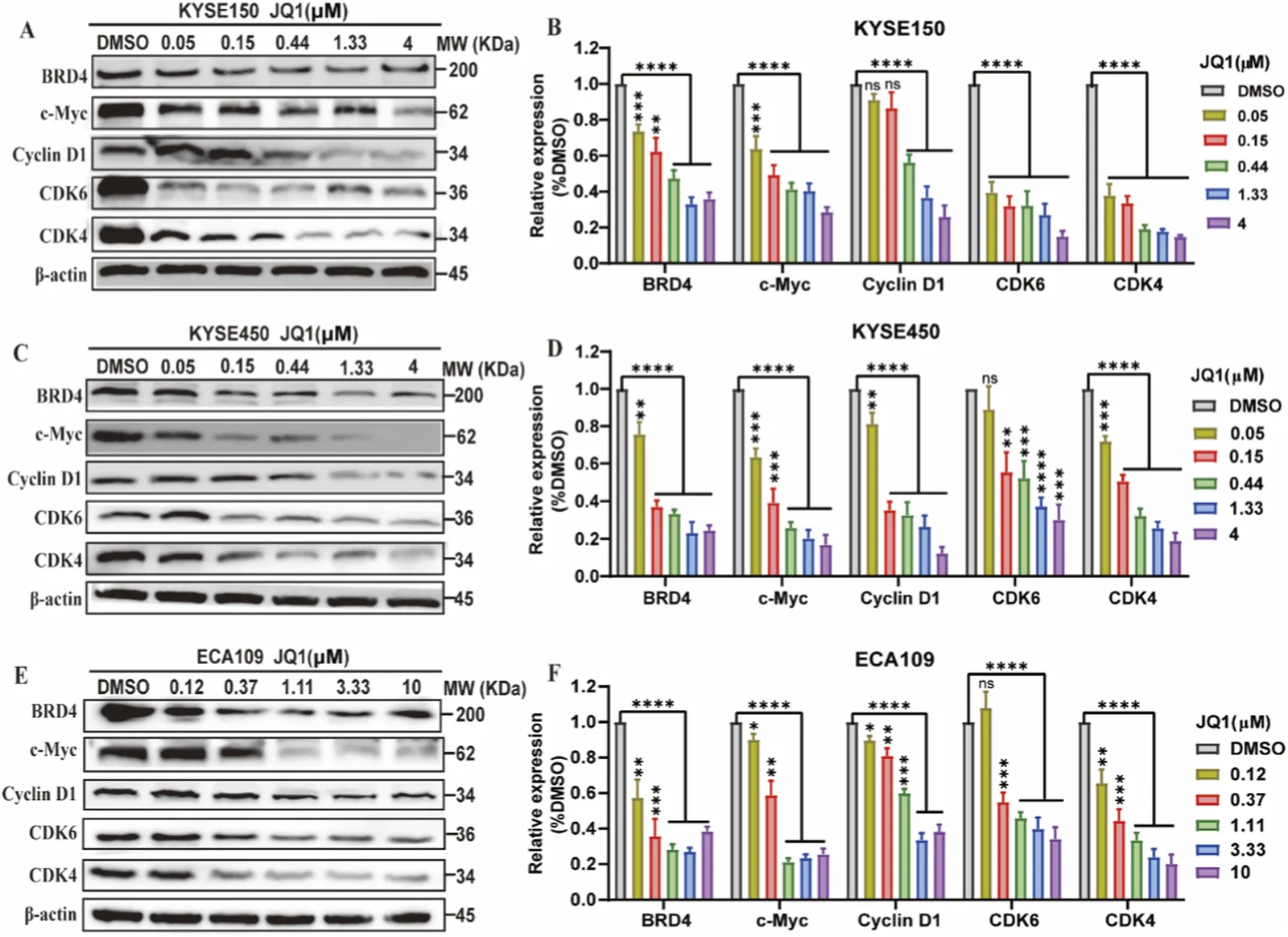
JQ1 modulates BRD4-associated G1/S regulatory signaling in ESCC cells. **(A)** Western blot analysis of BRD4, c-Myc, CDK4, CDK6, and Cyclin D1 expression in KYSE150 cells. **(B)** Quantitative analysis of the Western blot data shown in **(A)**. **(C)** Western blot analysis of BRD4, c-Myc, CDK4, CDK6, and Cyclin D1 expression in KYSE450 cells. **(D)** Quantitative analysis of the Western blot data shown in **(C)**. **(E)** Western blot analysis of BRD4, c-Myc, CDK4, CDK6, and Cyclin D1 expression in ECA109 cells. **(F)** Quantitative analysis of the Western blot data shown in **(E)**. Cells were treated with JQ1 at the indicated concentrations (0–10 μM) or vehicle control (DMSO) for 24 h β-actin was used as the loading control, and target protein levels were quantified and normalized to β-actin. Data are presented as the mean ± SD from three independent biological experiments. Statistical significance was assessed by one-way ANOVA followed by Tukey’s multiple comparisons test. *P < 0.05, **P < 0.01, ***P < 0.001, ****P < 0.0001; ns, not significant vs. DMSO control.

JQ1 treatment reduced BRD4 protein expression in KYSE150, KYSE450, and ECA109 cells (Figure 6). In parallel, the expression levels of c-Myc, Cyclin D1, CDK4, and CDK6 were decreased after JQ1 treatment, suggesting suppression of G1/S regulatory signaling. The magnitude of these protein changes varied among the three ESCC cell lines. KYSE150 and KYSE450 cells showed more evident reductions in BRD4 and cell-cycle-related proteins at relatively lower JQ1 concentrations, whereas ECA109 cells required higher concentrations to show comparable changes (Figures 6A–F). These molecular alterations were consistent with the cell line-dependent changes in cell-cycle distribution observed by flow cytometry. Together, these findings suggest that JQ1-induced G1-phase accumulation is accompanied by reduced BRD4 expression and downregulation of c-Myc/Cyclin D1/CDK4/6-related signaling in ESCC cells.

### 3.6 JQ1 promotes apoptosis in ESCC cells

**FIGURE 7:**
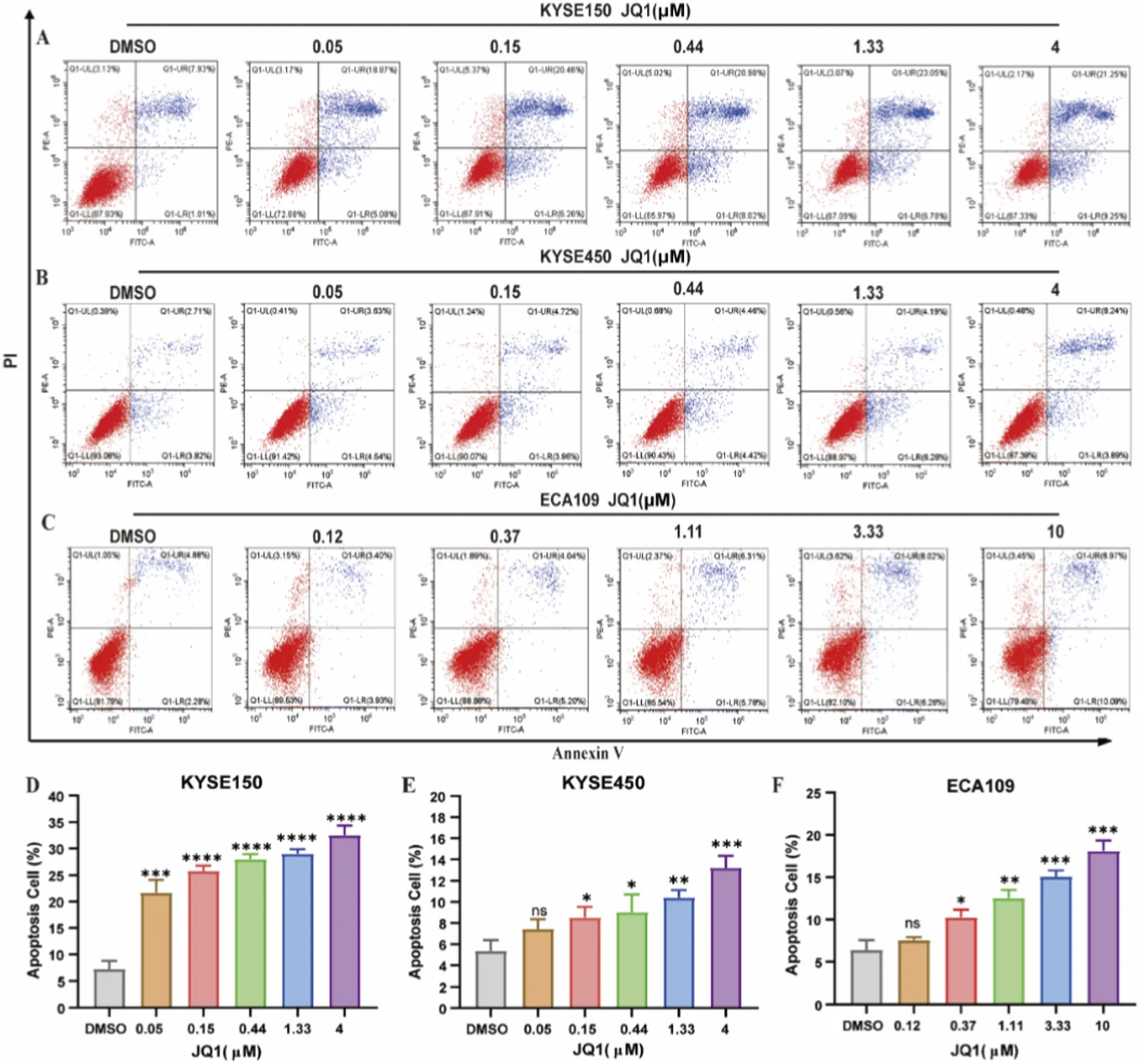
JQ1 promotes apoptosis in ESCC cells in a dose-dependent manner. **(A)** Representative flow cytometry plots showing apoptotic cells in KYSE150 cells. **(B)** Representative flow cytometry plots showing apoptotic cells in KYSE450 cells. **(C)** Representative flow cytometry plots showing apoptotic cells in ECA109 cells. **(D)** Quantification of apoptotic rates (% of total cells) from the data shown in **(A)**. **(E)** Quantification of apoptotic rates (% of total cells) from the data shown in **(B)**. **(F)** Quantification of apoptotic rates (% of total cells) from the data shown in **(C)**. Cells were treated with JQ1 at the indicated concentrations (0–10 μM) or vehicle control (DMSO) for 48 h, followed by Annexin V-FITC/PI staining and flow cytometry. Data are presented as the mean ± SD from three independent biological experiments. Statistical significance was assessed by one-way ANOVA followed by Tukey’s multiple comparisons test. *P < 0.05, **P < 0.01, ***P < 0.001, ****P < 0.0001; ns, not significant vs. DMSO control.

Flow cytometry with Annexin V-FITC/PI staining was performed to evaluate apoptosis after JQ1 treatment. A concentration-dependent increase in apoptotic cells was observed across the three ESCC cell lines, although the magnitude of the response differed among cell lines (Figure 7). KYSE150 cells showed a greater apoptotic response, with the total apoptotic fraction increasing to 32.52% ± 2.02% after treatment with 10 μM JQ1 (Figures 7A,D). In KYSE450 cells, 10 μM JQ1 increased the apoptotic rate from 5.37% ± 0.78% to 13.23% ± 1.10% (Figures 7B,E). In ECA109 cells, the apoptotic rate increased from 6.41% ± 1.33% to 18.09% ± 1.03% under the same high-dose condition (Figures 7C,F). These data indicate that JQ1 promotes apoptosis in ESCC cells, with cell line-dependent differences in sensitivity.

### 3.7 JQ1 modulates intrinsic apoptosis-related proteins in ESCC cells

**FIGURE 8:**
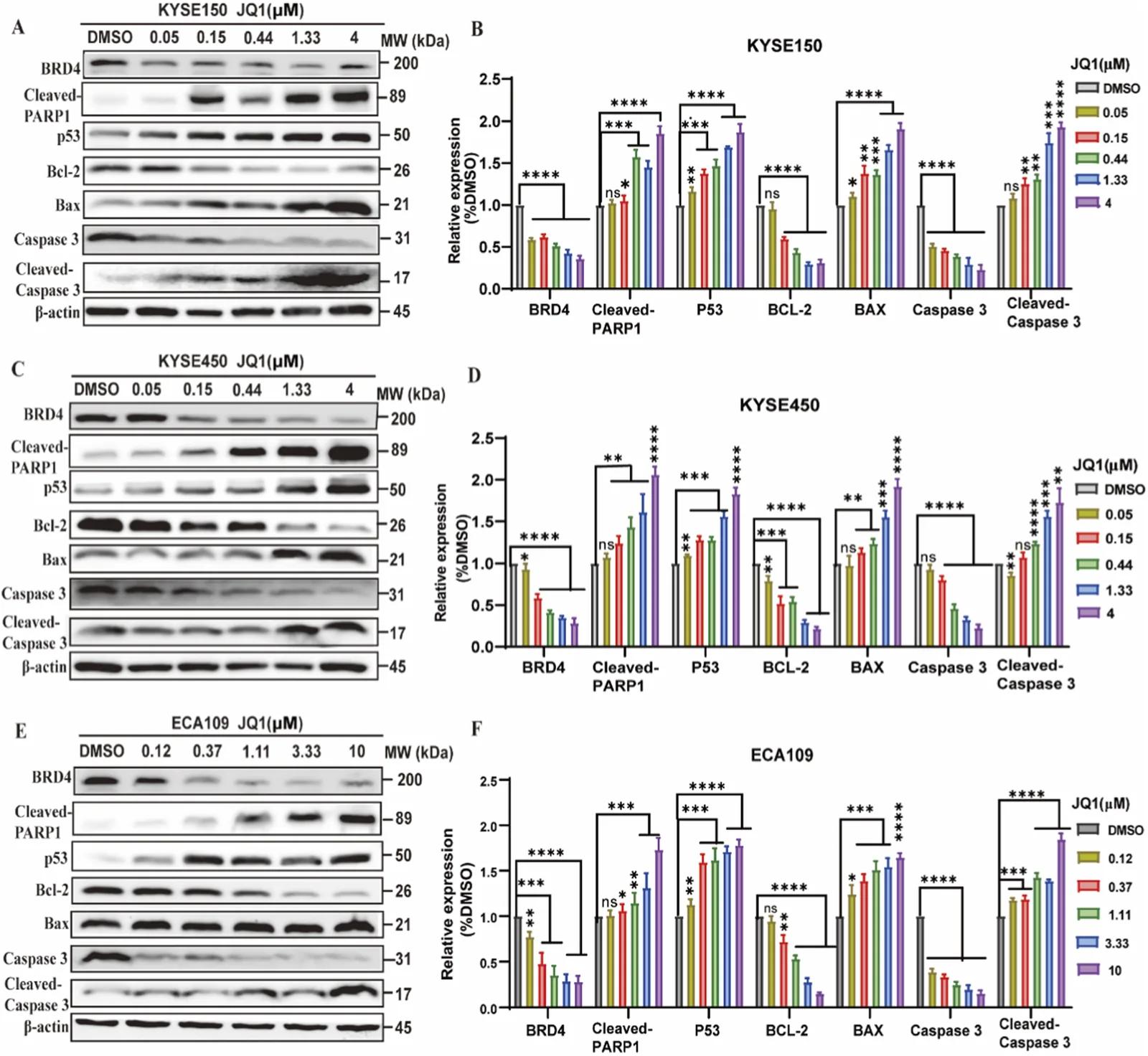
Western blot analysis of apoptosis-related proteins in ESCC cells following JQ1 treatment. **(A)** Expression levels of BRD4, cleaved PARP1, p53, Bcl-2, Bax, Caspase-3, and cleaved Caspase-3 in KYSE150 cells. **(B)** Quantitative analysis of the Western blot results shown in **(A)**. **(C)** Expression levels of BRD4, cleaved PARP1, p53, Bcl-2, Bax, Caspase-3, and cleaved Caspase-3 in KYSE450 cells. **(D)** Quantitative analysis of the Western blot results shown in **(C)**. **(E)** Expression levels of BRD4, cleaved PARP1, p53, Bcl-2, Bax, Caspase-3, and cleaved Caspase-3 in ECA109 cells. **(F)** Quantitative analysis of the Western blot results shown in **(E)**. Cells were treated with JQ1 at the indicated concentrations (0–10 μM) or vehicle control (DMSO) for 24 h β-actin was used as the loading control, and target protein levels were quantified and normalized to β-actin. Data are presented as the mean ± SD from three independent biological experiments. Statistical significance was assessed by one-way ANOVA followed by Tukey’s multiple comparisons test. *P < 0.05, **P < 0.01, ***P < 0.001, ****P < 0.0001; ns, not significant vs. DMSO control.

To further characterize the molecular changes associated with JQ1-induced apoptosis, Western blot analysis was performed to examine BRD4 and key apoptosis-related proteins in KYSE150, KYSE450, and ECA109 cells. JQ1 treatment reduced BRD4 protein expression in all three ESCC cell lines in a concentration-dependent manner. This reduction was accompanied by increased expression of p53 and Bax, together with decreased expression of the anti-apoptotic protein Bcl-2 (Figure 8). Because the Bax/Bcl-2 balance is closely associated with intrinsic mitochondrial apoptotic signaling (Huang et al., 2022; Zhang et al., 2021), these changes suggest that JQ1 treatment shifts ESCC cells toward a pro-apoptotic state.

To further assess apoptotic execution, the cleaved forms of apoptosis-related proteins were examined. JQ1 treatment increased the levels of cleaved PARP1 and cleaved Caspase-3, while reducing the level of full-length Caspase-3 (Figure 8). These changes are consistent with activation of the apoptosis-associated proteolytic cascade. The magnitude of these molecular alterations differed among the three ESCC cell lines. In KYSE150 and KYSE450 cells, JQ1 decreased BRD4 and Bcl-2 expression and increased p53, Bax, cleaved PARP1, and cleaved Caspase-3 within the tested concentration range of 0.05–4 μM (Figures 8A–D). In ECA109 cells, similar apoptosis-associated changes were observed over a broader concentration range of 0.12–10 μM, consistent with the relatively lower sensitivity of this cell line to JQ1 treatment (Figures 8E,F).

These protein expression changes were consistent with the apoptosis results obtained by flow cytometry, supporting that JQ1 treatment is associated with intrinsic apoptosis-related molecular alterations in ESCC cells. Although p53 expression increased after JQ1 treatment, the present data do not establish a direct regulatory relationship between BRD4 reduction and p53 activation. Therefore, p53 upregulation should be interpreted as an apoptosis- and stress-associated molecular change rather than evidence of direct BRD4-mediated p53 regulation.

### 3.8 JQ1 induces γ-H2AX accumulation and DNA strand breaks in ESCC cells

**FIGURE 9:**
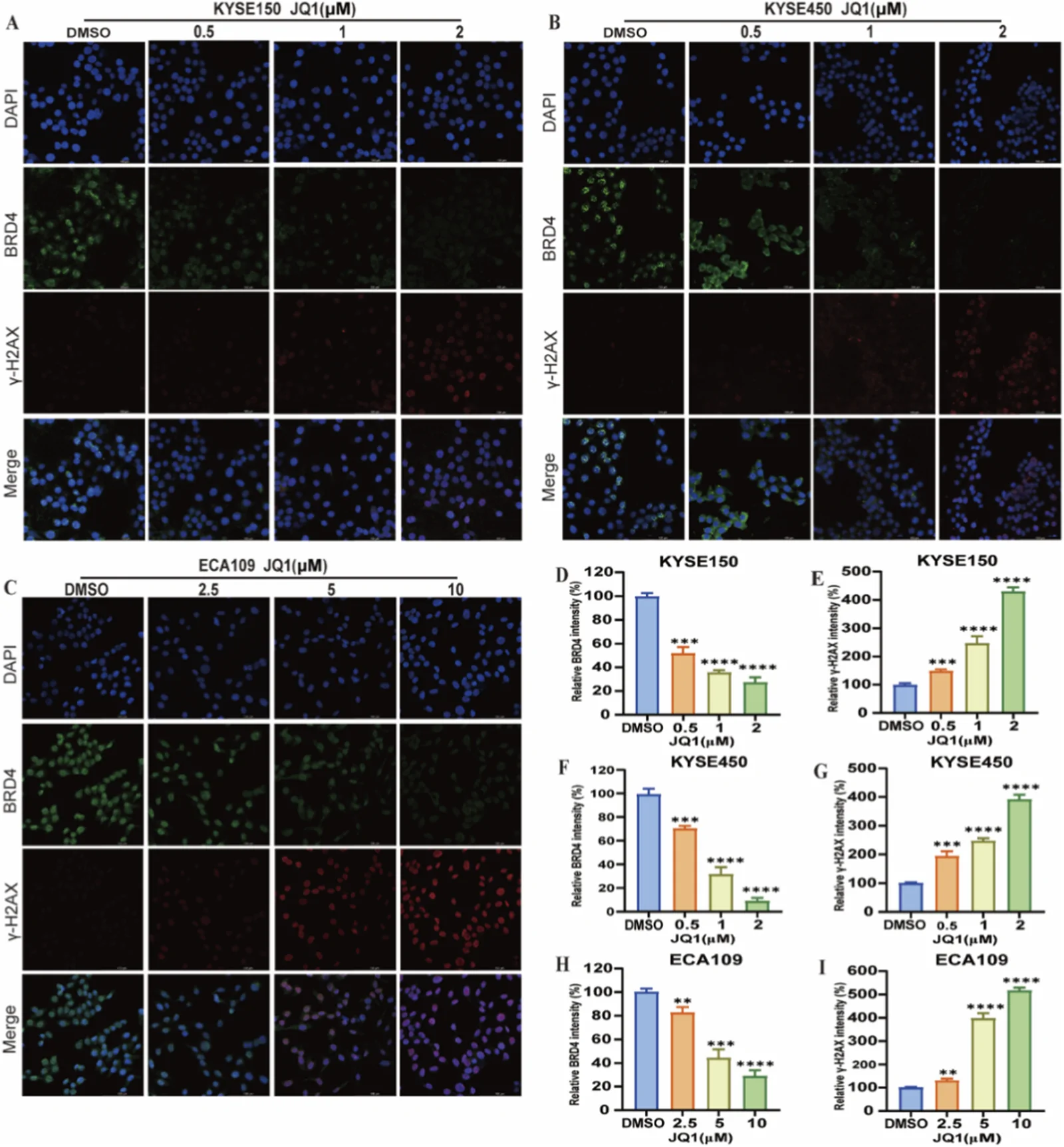
Immunofluorescence analysis of BRD4 and γ-H2AX expression in ESCC cells following JQ1 treatment. **(A)** Representative immunofluorescence images showing BRD4 and γ-H2AX expression in KYSE150 cells. **(B)** Representative immunofluorescence images showing BRD4 and γ-H2AX expression in KYSE450 cells. **(C)** Representative immunofluorescence images showing BRD4 and γ-H2AX expression in ECA109 cells. Cells were treated with JQ1 at the indicated concentrations (0–10 μM) or vehicle control (DMSO) for 48 h. Cells were stained with antibodies against BRD4 (green) and γ-H2AX (red), and nuclei were counterstained with DAPI (blue). Scale bar = 100 μm. **(D)** Quantification of BRD4 fluorescence intensity in KYSE150 cells. **(E)** Quantification of γ-H2AX fluorescence intensity in KYSE150 cells. **(F)** Quantification of BRD4 fluorescence intensity in KYSE450 cells. **(G)** Quantification of γ-H2AX fluorescence intensity in KYSE450 cells. **(H)** Quantification of BRD4 fluorescence intensity in ECA109 cells. **(I)** Quantification of γ-H2AX fluorescence intensity in ECA109 cells. Data are presented as the mean ± SD from three independent biological experiments. Statistical significance was assessed by one-way ANOVA followed by Tukey’s multiple comparisons test. *P < 0.05, **P < 0.01, ***P < 0.001, ****P < 0.0001; ns, not significant vs. DMSO control.

Because DNA damage is an important intracellular stress signal associated with apoptosis (Vodicka et al., 2025), we further examined whether JQ1 treatment was accompanied by activation of DNA damage-related responses in ESCC cells. Immunofluorescence staining was performed to detect BRD4 and γ-H2AX, the phosphorylated form of H2AX at serine 139 and a widely used marker of DNA damage response activation (Prabhu et al., 2024). In KYSE150 and KYSE450 cells, JQ1 treatment at 0.5, 1, and 2 μM reduced BRD4-associated green fluorescence and increased γ-H2AX-positive nuclear signals compared with the DMSO control group (Figures 9A,B). In ECA109 cells, similar changes were observed after treatment with higher concentrations of JQ1 at 2.5, 5, and 10 μM (Figure 9C).

Quantitative fluorescence analysis further supported these observations. BRD4 fluorescence intensity decreased significantly after JQ1 treatment in KYSE150, KYSE450, and ECA109 cells, whereas γ-H2AX fluorescence intensity increased in a concentration-dependent manner (Figures 9D–I). The increase in γ-H2AX was observed at relatively lower JQ1 concentrations in KYSE150 and KYSE450 cells, while ECA109 cells required higher concentrations to show comparable BRD4 reduction and γ-H2AX accumulation. This pattern was consistent with the differential sensitivity of these ESCC cell lines to JQ1 observed in the preceding assays.

**FIGURE 10:**
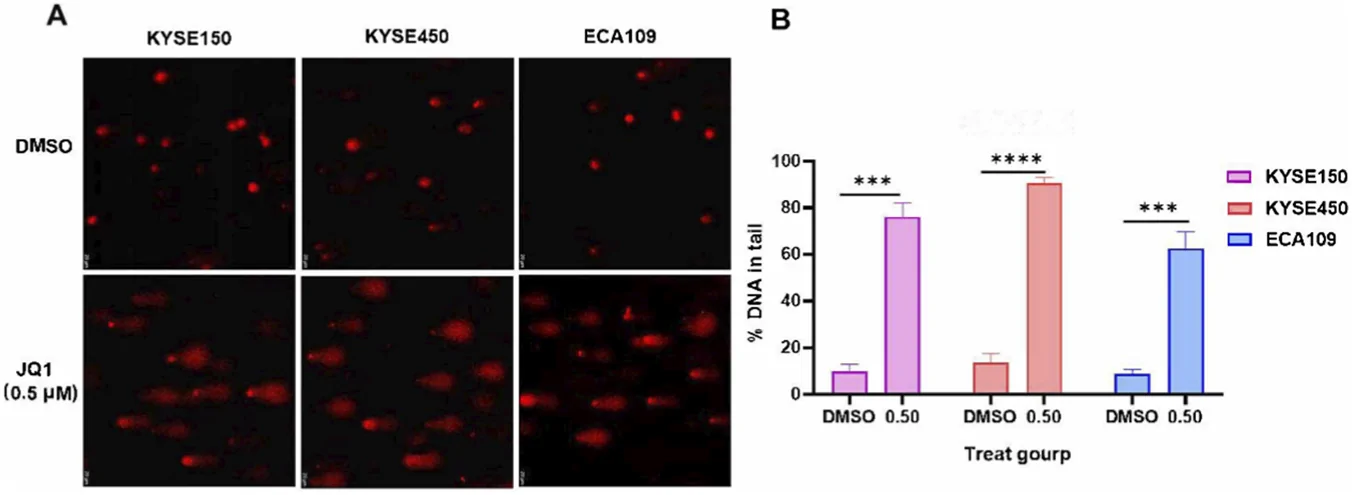
Alkaline comet assay shows increased DNA strand breaks after JQ1 treatment in ESCC cells. **(A)** Representative fluorescence images of alkaline comet assay in KYSE150, KYSE450, and ECA109 cells treated with DMSO or JQ1 at 0.5 μM for 24 h. **(B)** Quantitative analysis of DNA damage expressed as the percentage of DNA in the comet tail. Data are presented as the mean ± SD from three independent biological experiments. At least 50 cells were analyzed for each sample. Statistical significance was assessed by unpaired Student’s t-test between the DMSO and JQ1-treated groups within each cell line. ***P < 0.001, ****P < 0.0001 vs. DMSO control.

To further evaluate DNA strand breaks, an alkaline comet assay was performed in KYSE150, KYSE450, and ECA109 cells. Compared with DMSO-treated cells, JQ1-treated cells showed increased comet tail formation in all three ESCC cell lines (Figure 10A). Quantitative analysis showed that the percentage of DNA in the comet tail was significantly increased after JQ1 treatment (Figure 10B), indicating increased DNA strand breakage under alkaline electrophoresis conditions.

Taken together, these results show that JQ1 treatment is associated with reduced BRD4 expression, increased γ-H2AX accumulation, and enhanced DNA strand breaks in ESCC cells. These findings support the presence of DNA damage-associated cellular stress after JQ1 exposure, which is consistent with the observed cell-cycle arrest and apoptosis-related changes.

### 3.9 JQ1 suppresses KYSE150 xenograft growth in BALB/c nude mice

**FIGURE 11:**
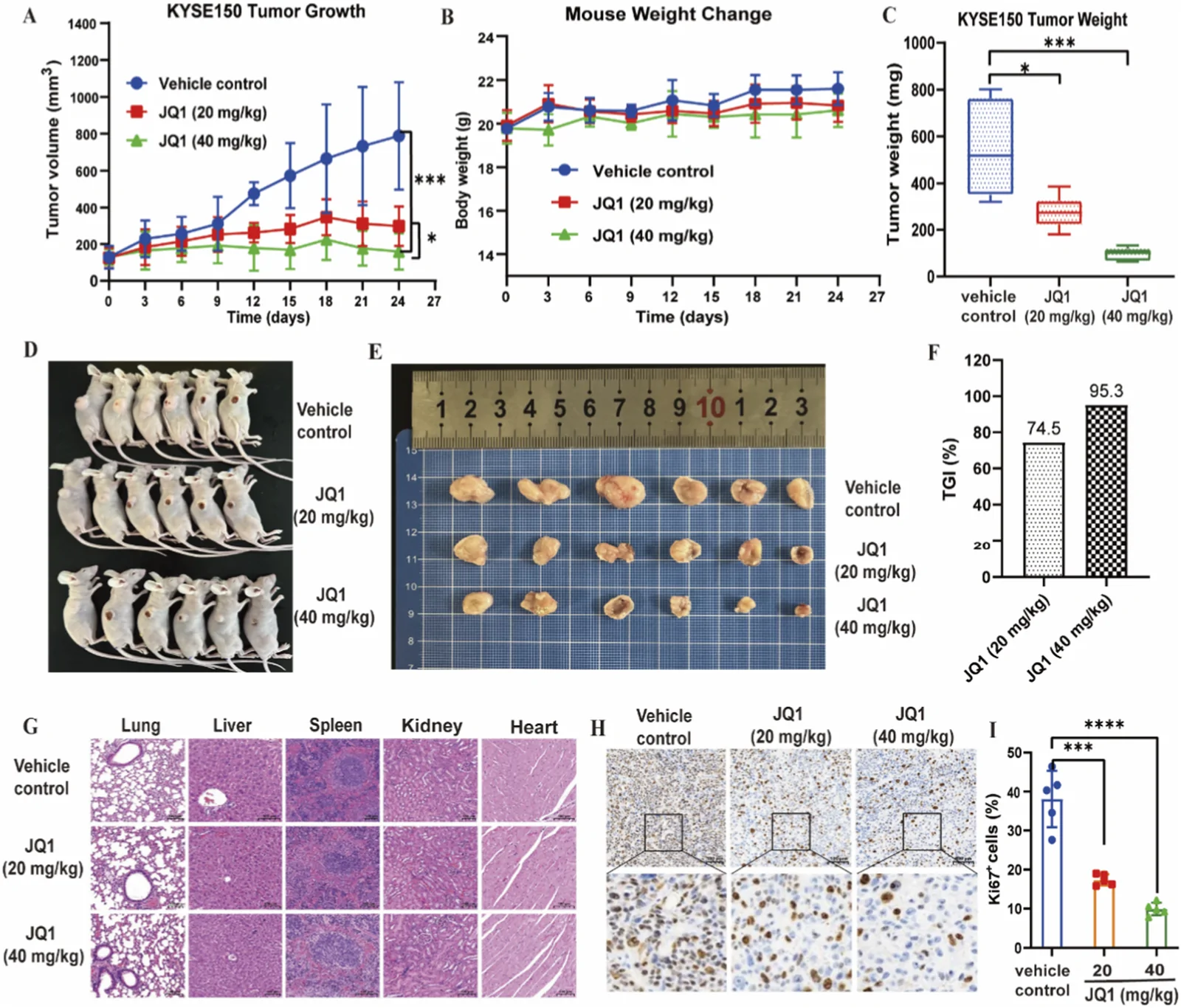
JQ1 suppresses KYSE150 xenograft growth in BALB/c nude mice. **(A)** Tumor growth curves of KYSE150 xenografts in mice treated with vehicle control, JQ1 at 20 mg/kg, or JQ1 at 40 mg/kg. **(B)** Body weight changes during the treatment period. **(C)** Final tumor weights of excised KYSE150 xenografts at the experimental endpoint. **(D)** Representative macroscopic images of xenograft-bearing mice from each group. **(E)** Representative images of excised tumors. **(F)** Tumor growth inhibition rates calculated for the JQ1-treated groups. **(G)** Representative H&E staining images of major organs, including lung, liver, spleen, kidney, and heart. Scale bar = 100 μm. **(H)** Representative immunohistochemical staining images showing Ki67 expression in xenograft tumor tissues. **(I)** Quantitative analysis of Ki67-positive cells in tumor sections. Data are presented as the mean ± SD; n = 6 mice per group, with each mouse considered one independent biological replicate. For panels A and B, statistical significance was assessed by two-way repeated-measures ANOVA followed by Tukey’s multiple comparisons test. For panels C and I, statistical significance was assessed by one-way ANOVA followed by Tukey’s multiple comparisons test. *P < 0.05, ***P < 0.001, ****P < 0.0001 vs. vehicle control.

The *in vivo* anti-tumor activity of JQ1 was evaluated using a KYSE150 subcutaneous xenograft model established in female BALB/c nude mice. Tumor-bearing mice were treated with vehicle control, 20 mg/kg JQ1, or 40 mg/kg JQ1. Compared with the vehicle control group, JQ1 treatment significantly suppressed xenograft growth, as shown by reduced tumor volume and final tumor weight (Figures 11A,C–E). The calculated tumor growth inhibition (TGI) rates were 74.5% and 95.3% in the 20 mg/kg and 40 mg/kg JQ1 groups, respectively (Figure 11F), suggesting a dose-related tumor growth-suppressive effect *in vivo*.

Mouse body weight was monitored throughout the treatment period as a preliminary indicator of systemic tolerability. No obvious body weight loss was observed in either JQ1-treated group compared with the vehicle control group (Figure 11B). In addition, H&E staining of major organs, including the lung, liver, spleen, kidney, and heart, did not reveal apparent histopathological abnormalities under the current experimental conditions (Figure 11G). These observations suggest that JQ1 treatment did not induce overt toxicity detectable by body weight monitoring or routine histological examination in this xenograft model.

To assess the effect of JQ1 on tumor cell proliferation *in vivo*, Ki67 immunohistochemistry was performed on xenograft tumor sections. JQ1 treatment reduced the percentage of Ki67-positive cells in KYSE150 xenografts, with a greater reduction observed in the 40 mg/kg group than in the 20 mg/kg group (Figures 11H,I). These results indicate that JQ1 suppresses KYSE150 xenograft growth and reduces tumor cell proliferation *in vivo*.

## 4 Discussion

**FIGURE 12:**
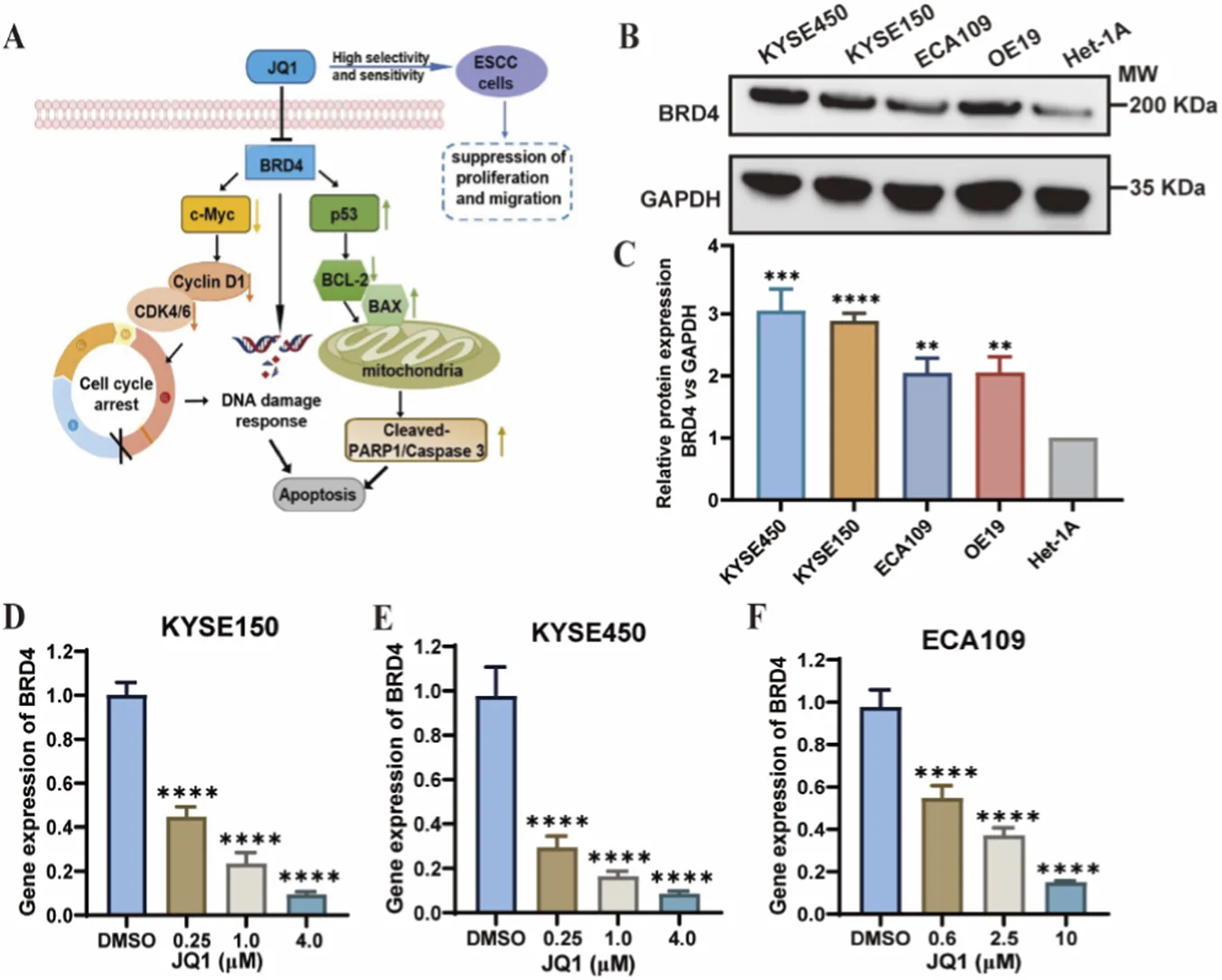
JQ1 treatment is associated with BRD4 suppression and downstream anti-tumor signaling changes in ESCC cells. **(A)** Schematic illustration of the proposed working model by which JQ1 may suppress ESCC cell proliferation and migration and promote DNA damage response and apoptosis. JQ1 treatment is associated with reduced BRD4 expression, inhibition of c-Myc/Cyclin D1-related cell-cycle signaling, increased p53 expression, modulation of the Bcl-2/Bax balance, and activation of cleaved PARP1/Caspase-3-associated apoptotic signaling. **(B)** Western blot analysis of basal BRD4 protein expression in KYSE450, KYSE150, ECA109, OE19, and Het-1A cells. GAPDH was used as the loading control. **(C)** Quantitative analysis of BRD4 protein expression normalized to GAPDH. **(D–F)** qRT-PCR analysis of BRD4 mRNA expression in KYSE150, KYSE450, and ECA109 cells after treatment with DMSO or the indicated concentrations of JQ1. Data are presented as the mean ± SD from three independent biological experiments. Statistical significance was assessed by one-way ANOVA followed by Tukey’s multiple comparisons test. **P < 0.01, ***P < 0.001, ****P < 0.0001 vs. Het-1A in panel **(C)** or vs. DMSO control in panels **(D–F)**.

In the present study, JQ1 showed anti-proliferative, pro-apoptotic, and anti-migratory effects in ESCC experimental models. Notably, the inhibitory effect on cell migration was observed at cell-line-specific low concentrations that did not significantly affect cell viability during the 24 h assay period. At the molecular level, JQ1 treatment was accompanied by reduced BRD4 expression, suppression of c-Myc/Cyclin D1-related signaling, activation of DNA damage-associated responses, and modulation of apoptosis-related proteins (Figure 12A). These findings suggest that JQ1 affects multiple proliferation-, stress-, and apoptosis-associated signaling events in ESCC cells.

BRD4 is an epigenetic reader protein that regulates transcriptional programs involved in tumor growth, survival, and cell-cycle progression (Wu et al., 2019; Zeng et al., 2024; Zhou et al., 2022). In this study, basal BRD4 protein expression was higher in ESCC cell lines than in the normal esophageal epithelial cell line Het-1A (Figures 12B,C). qRT-PCR analysis further showed that JQ1 treatment decreased BRD4 mRNA levels in KYSE150, KYSE450, and ECA109 cells in a dose-dependent manner (Figures 12D–F). Together with the observed reduction in BRD4 protein expression after JQ1 treatment, these data indicate that JQ1 is associated with suppression of BRD4 expression at both mRNA and protein levels in ESCC cells. However, because JQ1 functions as a BET bromodomain inhibitor and may influence broader BET-associated transcriptional networks (Lyu et al., 2025; To et al., 2023), these findings should be interpreted as JQ1-associated anti-tumor effects involving BRD4-related signaling changes, rather than evidence that these phenotypes are mediated only by BRD4-dependent mechanisms.

The DNA damage-related findings also require balanced interpretation. γ-H2AX is widely used as a marker of DNA damage response activation, but its accumulation may also occur during replication stress or apoptosis (Fragkos et al., 2023; Prabhu et al., 2024). In this study, JQ1 increased γ-H2AX accumulation and enhanced comet tail formation in KYSE150, KYSE450, and ECA109 cells, supporting the presence of DNA damage-associated responses and DNA strand breaks after JQ1 exposure. The concomitant increase in p53 expression may therefore reflect, at least in part, a cellular response to JQ1-induced DNA damage or broader stress signaling (Dong et al., 2018; Xu et al., 2020). Nevertheless, the current data do not define the precise temporal sequence among BET/BRD4-associated signaling changes, DNA damage response activation, p53 upregulation, cell-cycle arrest, and apoptosis.

JQ1 also reduced ESCC cell migration under non-cytotoxic conditions, as demonstrated by wound healing and Transwell assays performed with cell-line-specific low-dose ranges and parallel viability controls. This experimental design minimized the possibility that reduced migration was merely a secondary consequence of short-term cytotoxicity. The accompanying changes in c-Myc/Cyclin D1 signaling and EMT-related markers were consistent with the observed reduction in cell motility. However, previous studies have reported context-dependent effects of JQ1 on EMT, autophagy, and migration (Lu et al., 2020; Wang J. et al., 2017; Wen et al., 2020; Yang et al., 2022), suggesting that the molecular basis of JQ1-regulated migration in ESCC may depend on cellular context and requires further clarification.

The *in vivo* xenograft experiment further supported the tumor growth-suppressive activity of JQ1 in ESCC. JQ1 treatment reduced KYSE150 xenograft volume and tumor weight and decreased Ki67 expression, indicating reduced tumor cell proliferation *in vivo*. Body weight monitoring and routine H&E staining of major organs did not reveal obvious toxicity under the current experimental conditions. These observations provide preliminary tolerability evidence in this xenograft model, although more comprehensive safety evaluation will be needed in future studies. Because tumor-tissue analysis focused mainly on Ki67, the xenograft results primarily support an anti-proliferative effect *in vivo*, whereas the contribution of apoptosis and DNA damage to the *in vivo* response remains to be further investigated.

Overall, this study provides a controlled preclinical evaluation of JQ1 in ESCC. The findings support the ability of JQ1 to suppress ESCC cell growth and migration and to induce apoptosis- and DNA damage-associated molecular changes. While BRD4-related signaling appears to be involved, further target-specific validation and expanded *in vivo* mechanistic studies are needed to more clearly define the therapeutic potential of BET/BRD4-associated pharmacological strategies in ESCC.

## 5 Conclusion

This study provides a controlled preclinical evaluation of JQ1 in ESCC experimental models. JQ1 displayed anti-proliferative, pro-apoptotic, and anti-migratory effects in ESCC cells, with migration inhibition observed at cell-line-specific low concentrations that did not significantly reduce cell viability. These effects were accompanied by reduced BRD4 expression, modulation of c-Myc/Cyclin D1-related cell-cycle signaling, DNA damage-associated responses, and apoptosis-related molecular changes. *In vivo*, JQ1 suppressed KYSE150 xenograft growth and reduced Ki67 expression, while body weight monitoring and routine histological assessment did not reveal obvious toxicity under the current experimental conditions. Collectively, these findings support further investigation of JQ1 and BET/BRD4-associated pharmacological strategies in ESCC. Further target-specific validation, expanded *in vivo* mechanistic studies, and broader safety evaluation are warranted to clarify its translational relevance.

## Data Availability

The original contributions presented in the study are included in the article/supplementary material, further inquiries can be directed to the corresponding authors.
